# A Phase I study of pazopanib in combination with gemcitabine in patients with advanced solid tumors

**DOI:** 10.1007/s00280-012-1982-z

**Published:** 2012-10-11

**Authors:** Ruth Plummer, Ayman Madi, Melinda Jeffels, Heike Richly, Bahar Nokay, Stephen Rubin, Howard A. Ball, Steve Weller, Jeffrey Botbyl, Diana M. Gibson, Max E. Scheulen

**Affiliations:** 1 Sir Bobby Robson Cancer Trials Research Centre, Northern Centre for Cancer Care, Newcastle upon Tyne, NE7 7DN UK; 2 Department of Medical Oncology, West German Cancer Center, University Hospital Essen, University of Duisburg-Essen, Essen, Germany; 3 GlaxoSmithKline, Collegeville, PA USA; 4 Present Address: Astellas, Deerfield, IL USA; 5 GlaxoSmithKline, Research Triangle Park, NC USA; 6 Provonix, Mullica Hill, NJ USA

**Keywords:** Anti-angiogenesis, Combination therapy, Gemcitabine, Melanoma, Pazopanib, Pharmacokinetics, Phase I, Solid tumors

## Abstract

**Purpose:**

Pazopanib plus gemcitabine combination therapy was explored in patients with advanced solid tumors.

**Methods:**

In a modified 3 + 3 enrollment scheme, oral once-daily pazopanib was administered with intravenous gemcitabine (Days 1 and 8, 21-day cycles). Three protocol-specified dose levels were tested: pazopanib 400 mg plus gemcitabine 1,000 mg/m^2^, pazopanib 800 mg plus gemcitabine 1,000 mg/m^2^, and pazopanib 800 mg plus gemcitabine 1,250 mg/m^2^. Maximum-tolerated dose was based on dose-limiting toxicities during treatment Cycle 1. In the expansion phase, six additional patients were enrolled at the highest tolerable dose level.

**Results:**

Twenty-two patients were enrolled. At the highest dose level tested (pazopanib 800 plus gemcitabine 1,250), patients received >80 % of their planned dose and the regimen was deemed safe and tolerable. The most common treatment-related adverse events included fatigue, neutropenia, nausea, and decreased appetite. Neutropenia and thrombocytopenia were the most common events leading to dose modifications. Pharmacokinetic interaction between pazopanib and gemcitabine was not observed. One objective partial response at the highest dose was observed in a patient with metastatic melanoma. Prolonged disease stabilization (>12 cycles) was reported in three patients (metastatic melanoma, cholangiocarcinoma, and colorectal carcinoma).

**Conclusion:**

Combination pazopanib plus gemcitabine therapy is tolerable, with an adverse event profile reflective of that associated with the individual agents. There was no apparent pharmacokinetic interaction with pazopanib plus gemcitabine co-administration, although patient numbers were limited. Further investigation of combined pazopanib plus gemcitabine is warranted.

## Introduction

Pazopanib is a multi-tyrosine kinase inhibitor of vascular endothelial growth factor receptor (VEGFR)-1, VEGFR-2, VEGFR-3, platelet-derived growth factor receptor (PDGFR)-α, PDGFR-β, fibroblast growth factor receptor (FGFR)-1, FGFR-3, and c-Kit. Pazopanib is approved as monotherapy for patients with advanced renal cell carcinoma [1] and soft tissue sarcoma [2] and is currently under investigation in multiple tumor types, including ovarian cancer, non-small-cell lung cancer, thyroid cancer, and cervical cancer [3–7].

Gemcitabine is a cytotoxic nucleoside analogue of deoxycytidine whose triphosphate (dFdCTP) is irreversibly incorporated into DNA, subsequently inhibiting exonuclease and DNA repair activities. Gemcitabine has broad-spectrum activity and is approved or commonly used, either as a single agent or in combination with other chemotherapy agents, for the treatment of ovarian cancer, breast cancer, non-small-cell lung cancer, pancreatic cancer, and soft tissue sarcoma [8–12]. Myelosuppression was the dose-limiting toxicity (DLT) in gemcitabine single-agent Phase I trials [13, 14].

Clinical studies exploring therapeutic strategies that combine angiogenesis pathway inhibition with concurrent chemotherapy have shown promise for the treatment of various malignancies [15–17]. Therefore, a Phase I study (NCT00678977; VEG109599) was conducted to determine the maximum-tolerated dose (MTD) of pazopanib in combination with gemcitabine. Secondary objectives included evaluation of safety and pharmacokinetics of the combination and assessment of the preliminary clinical activity in patients with advanced solid tumors.

## Patients and methods

### Study participants

Eligible patients were at least 18 years of age with a histologically or cytologically confirmed advanced solid tumor, who had progressed on standard therapy or for whom no standard therapy was available. Additional eligibility criteria included an Eastern Cooperative Oncology Group (ECOG) performance status of 0 or 1; measurable or evaluable disease at the time of screening; adequate hematologic, hepatic, and renal function; and no unstable or serious concurrent medical condition. An unlimited number of prior therapies were permitted; however, at least 4 weeks must have elapsed since previous treatment. Patients with asymptomatic brain metastases who did not require steroids and antiseizure medications for more than 3 months were eligible.

Exclusion criteria included the presence of leptomeningeal carcinomatosis; clinically significant gastrointestinal abnormality; elevated blood pressure (≥140/90 mmHg); prolonged QT interval (>480 ms); history of cardiac angioplasty or stenting, myocardial infarction, unstable angina, symptomatic peripheral vascular disease, or Class III or IV congestive heart failure; uncontrolled infection; history of cerebrovascular accident, pulmonary embolism, or untreated deep vein thrombosis within the previous 6 months; and previous treatment with an investigational or licensed tyrosine kinase inhibitor targeting VEGF receptors.

The study was conducted in accordance with the standards of each site’s independent ethics committees, principles of good clinical practice, all applicable regulatory requirements, and the Declaration of Helsinki. All patients provided written informed consent before enrollment and before undergoing any study-specific procedures.

### Study design

This open-label study consisted of a dose-escalation phase to determine the MTD and a fixed-dose, cohort-expansion phase to further define the safety and tolerability of the MTD. The dose-escalation phase used a 3 + 3 enrollment design. Initially, three patients were enrolled into Dose Level 1; if no DLT was observed, three patients were enrolled at the next dose level. If a DLT was observed in one of the first three patients enrolled at a given dose level, three additional patients were enrolled at that dose level. Escalation to the subsequent dose level was permitted if no more than one of six patients experienced a DLT. If, however, two or more patients experienced a DLT at a given dose level, the MTD was considered to have been exceeded and a lower or intermediate dose level would be explored.

Dose-limiting toxicity was based on observed toxicity during the first cycle of treatment. Toxicities included the following: Grade 4 neutropenia, febrile neutropenia, Grade 4 anemia, platelet count below 25,000, serum creatinine at least 2 times baseline or upper limit of normal, Grade 3 proteinuria with uncontrolled hypertension or renal impairment and Grade 4 proteinuria, Grade 3 or higher non-hematologic toxicity (except fatigue but including nausea, vomiting, and diarrhea not controlled by supportive treatment), Grade 3 uncontrolled hypertension, Grade 4 hypertension, delay of treatment for more than 3 weeks, or inability to receive 75 % of scheduled doses in a treatment cycle.

### Treatment

During the dose-escalation phase, 3 protocol-defined dose regimens were evaluated. In this phase, pazopanib was administered orally once daily beginning on Day 1 of Cycle 1, and gemcitabine was administered intravenously on Day 1 and Day 8 of each 21-day cycle. In the cohort-expansion phase, gemcitabine was administered on the same schedule, but daily pazopanib dosing began on Day 2 of Cycle 1 to permit determination of gemcitabine-alone pharmacokinetics on Day 1. Gemcitabine infusions were administered over 30 min. Intrapatient dose escalations were not permitted. Dose modifications and reductions for pazopanib were to be performed for the control of blood pressure or in the event of hemorrhage, thrombosis, proteinuria, or hepatotoxicity. Dose modifications and reductions for gemcitabine were based on hematologic toxicity and other toxicities causally related to gemcitabine. Treatment could continue in the absence of unacceptable toxicities, disease progression, patient withdrawal of consent, investigator decision, or a delay in treatment for more than 3 weeks.

### Patient evaluation

Screening assessments were completed within 28 days before the first dose of study treatment; these included medical history, prior anticancer therapy, physical examination, ECOG performance status, vital signs, hematology, and clinical chemistry. Baseline electrocardiogram and echocardiogram or multi-gated acquisition (MUGA) scans were also performed.

Safety was assessed throughout the study by physical examination, 12-lead electrocardiograms, echocardiograms or MUGA, vital sign measurements, and clinical laboratory tests. Patients were monitored for adverse events (AEs) throughout the study. The frequency, severity, and relationship to treatment for AEs that occurred during study treatment and up to 30 days after the last dose of study drug were evaluated. Adverse events were coded according to the Medical Dictionary for Regulatory Activities (MedDRA) and Common Terminology Criteria for Adverse Events (CTCAE) version 3.0. All patients who received at least one dose of study drug were included in the safety analyses.

Disease assessment was performed within 28 days before the first dose of study treatment and every 2 treatment cycles thereafter. All patients completing at least 2 treatment cycles were evaluable for response. Tumor response was evaluated according to Response Evaluation Criteria in Solid Tumors [18]. Confirmatory scans were required at least 4 weeks after the initial documentation of a complete or partial response.

### Pharmacokinetic sampling

Sparse sampling was performed during the dose-escalation phase; serial blood samples for plasma pazopanib analysis were nominally collected pre-dose and 3.5 h post-dose on Day 1 of Cycle 1, and pre-dose on Day 8 of Cycle 1 and Day 1 of Cycle 2. Blood samples for analysis of plasma gemcitabine and its metabolite, 2′,2′-difluorodeoxyuridine (dFdU), were nominally collected pre-dose and at 0.5 h (i.e., at the end of gemcitabine infusion) on Days 1 and 8 of Cycle 1. In the cohort-expansion phase, blood samples for plasma gemcitabine and dFdU analysis were collected pre-dose and at 0.25, 0.5, 1, 1.5, 2, 4, 6, 8, and 24 h after the start of the gemcitabine infusion on Day 1 of Cycle 1 (gemcitabine alone) and at the same times on Day 1 of Cycle 2 (gemcitabine and pazopanib in combination) for gemcitabine, dFdU, and pazopanib analysis.

### Drug concentration assays

Plasma samples were analyzed for pazopanib using a validated analytical method based on protein precipitation, followed by high-performance liquid chromatography/tandem mass spectrometry (HPLC/MS/MS) analysis [19]. The lower limit of quantification (LLQ) was 100 ng/mL, using a 20-μL aliquot of human plasma with a higher limit of quantification (HLQ) of 50,000 ng/mL. Plasma concentrations of gemcitabine and dFdU were determined using a validated method based on liquid/liquid extraction with methyl tertiary-butyl ether (MTBE) and chemical derivatization with dansyl chloride, followed by HPLC/MS/MS analysis. The LLQ for gemcitabine and dFdU was 50 and 500 ng/mL, respectively, using a 50-μL aliquot of human plasma with HLQ of 50,000 ng/mL for both gemcitabine and dFdU. For each assay, quality control (QC) samples, prepared at three different analyte concentrations and stored with study samples, were analyzed with each batch of samples against separately prepared calibration standards. For the analysis to be acceptable, no more than one-third of the QC results were to deviate from the nominal concentration by more than 15 %, and at least 50 % of the results from each QC concentration would be within 15 % of nominal.

### Statistical and pharmacokinetic analysis

Statistical analyses were performed using the SAS/STAT module of SAS, version 9 (SAS Institute, Cary, NC). Pharmacokinetic concentrations (dose-escalation and expansion cohorts) and pharmacokinetic parameters (expansion cohort only) for pazopanib, gemcitabine, and dFdU were summarized by dose cohort. In the expansion cohort, for each of the analytes, pharmacokinetic parameter estimates were obtained for maximum concentration (*C*_max_), time of *C*_max_, area under the plasma concentration–time curve (AUC) from time 0 to time post-dose of last quantifiable concentration, and elimination half-life. For gemcitabine, AUC extrapolated to infinity (AUC_(0–∞)_) and systemic clearance were estimated; AUC_(0–∞)_ was also estimated for dFdU. To assess the potential effect of pazopanib on gemcitabine pharmacokinetics, gemcitabine and dFdU pharmacokinetic parameters *C*_max_, AUC_(0–∞)_, and elimination half-life were log_e_-transformed and analyzed by mixed-effect analysis of variance (ANOVA), fitting terms for treatment (test: pazopanib plus gemcitabine [Cycle 2, Day 1]; reference: gemcitabine alone [Cycle 1, Day 1]) as a fixed effect and patient as a random effect. Geometric least squares means and 90 % confidence intervals (CI) for the differences in log_e_-transformed parameters were then back-transformed to obtain the geometric mean ratio (test/reference) and associated 90 % CI on the original scale.

Adverse events were listed and summarized by treatment regimen and the percent of patients reporting each event at least once. Laboratory parameters, vital signs, and electrocardiograms were summarized by time point and treatment regimen. Dose intensity was defined as ([actual dose/planned dose] × 100).

## Results

### Patient characteristics

Between April 2008 and March 2010, a total of 22 patients with advanced solid tumors were enrolled and treated with pazopanib plus gemcitabine; 21 patients completed the study. Two patients with melanoma who received pazopanib 800 mg plus gemcitabine 1,250 mg/m^2^ (Paz800/Gem1250) continued pazopanib treatment for an additional 14 and 16 months, respectively, after database lock. The most frequent tumor type was melanoma (eight patients, 36 %; Table 1). All patients had received at least 1 prior line of chemotherapy; 13 patients (59 %) had received at least 2 prior lines of chemotherapy.

**Table 1:** Patient demographics and disease characteristics at baseline

	Paz400/Gem1000 (*n* = 6)	Paz800/Gem1000 (*n* = 3)	Paz800/Gem1250 (*n* = 13)	Total (*N* = 22)
Gender (*n* %)				
Female	4 (67)	2 (67)	5 (38)	11 (50)
Male	2 (33)	1 (33)	8 (62)	11 (50)
Race (*n* %)				
White	6 (100)	3 (100)	13 (100)	22 (100)
Ethnicity (*n* %)				
Hispanic or Latino	0	0	0	0
Not Hispanic or Latino	6 (100)	3 (100)	13 (100)	22 (100)
Median age, years (range)	56 (22–63)	49 (47–52)	63 (30–74)	56 (22–74)
ECOG PS (*n* %)				
0	2 (33)	3 (100)	9 (69)	14 (64)
1	4 (67)	0	4 (31)	8 (36)
Primary tumor (*n* %)				
Melanoma	1 (17)	1 (33)	6 (46)	8 (36)
NSCLC	0	0	3 (23)	3 (14)
Colorectal	1 (17)	1 (33)	1 (8)	3 (14)
Cervix	1 (17)	0	0	1 (5)
Esophagus	0	0	1 (8)	1 (5)
Ovarian	1 (17)	0	0	1 (5)
Stomach	0	0	1 (8)	1 (5)
Other	2 (33)	0	1 (8)	3 (14)
Unknown	0	1 (33)	0	1 (5)

*ECOG PS* Eastern Cooperative Oncology Group performance status, *Gem* gemcitabine, *NSCLC* non-small-cell lung cancer, *Paz* pazopanib

### Dose escalation and determination of MTD

A DLT of Grade 4 thrombocytopenia was reported in one of the initial three patients (Table 2) enrolled in Dose Level 1 (pazopanib 400 mg plus gemcitabine 1,000 mg/m^2^ [Paz400/Gem1000]). As a result, Dose Level 1 was expanded to a total of six patients. No further DLTs were reported in Dose Level 1. No DLTs were observed in the dose-escalation phase of Dose Level 2 (pazopanib 800 mg plus gemcitabine 1,000 mg/m^2^ [Paz800/Gem1000]) or in the first three patients enrolled in Dose Level 3 (Paz800/Gem1250). Because Dose Level 3 was the highest protocol-defined dose level at which pazopanib and gemcitabine were administered at therapeutic levels equivalent to that of monotherapy with the individual agents, additional dose levels were not evaluated. Thus, the MTD was not determined. Overall, 1 DLT (Grade 3 fatigue) was reported among 13 patients enrolled in Dose Level 3 in the dose-escalation and cohort-expansion phase.

**Table 2:** Summary of exposure and dose-limiting toxicity

Dose level	Number of patients	Number of DLTs^a^	Median number of cycles (range)	
			Paz	Gem
Paz400/Gem1000	6	1^b^	5.5 (2–17)	5.5 (2–17)
Paz800/Gem1000	3	0	6 (5–12)	6 (5–12)
Paz800/Gem1250	13	1^c^	4 (1–14)	4 (1–14)

*DLT* dose-limiting toxicity, *Gem* gemcitabine, *Paz* pazopanib

^a^Observed during Cycles 1 and 2 during the dose-escalation phase

^b^Grade 4 thrombocytopenia

^c^Grade 3 fatigue

### Exposure

The median number of cycles of pazopanib and gemcitabine received in the Paz400/Gem1000 (*n* = 6), Paz800/Gem1000 (*n* = 3), and Paz800/Gem1250 (*n* = 13) dose levels were 5.5 cycles (range 2–17), 6 cycles (range 5–12), and 4 cycles (range 1–14), respectively. The median dose intensity for each of the three dose levels tested was as follows: Paz400/Gem1000, 99 %/78 %; Paz800/Gem1000, 95 %/78 %; and Paz800/Gem1250, 100 %/81 %. At least one pazopanib dose delay was observed in 12 patients (55 %), and 17 patients (77 %) had at least one gemcitabine dose delay. Across all dose levels, the most common AEs leading to a dose delay were neutropenia (three patients, 14 %), thrombocytopenia (2 patients, 9 %), and diarrhea (two patients, 9 %). Two patients (9 %) required at least one pazopanib dose reduction, whereas 12 patients (55 %) required at least one gemcitabine dose reduction. The most common AEs resulting in dose reductions were neutropenia (41 %), thrombocytopenia (14 %), and hypertension (5 %).

### Safety and tolerability

The most common treatment-related AEs reported in patients across all dose levels were fatigue (68 %), neutropenia (59 %), nausea (55 %), and decreased appetite (50 %; Table 3). The majority of treatment-related AEs were Grade 1 or 2. Seven patients (32 %) across all dose levels experienced Grade 4 treatment-related AEs of neutropenia and thrombocytopenia. The majority of patients (41 %) discontinued treatment due to disease progression; three patients (14 %) discontinued due to AEs (Grade 3 increased alanine aminotransferase, Grade 1 hematoma, and Grade 3 fatigue), two patients (9 %) discontinued at the investigator’s discretion, and four patients (18 %) withdrew consent. One treatment-related death (Grade 5 pneumonia) was reported in Dose Level 1 (Paz400/Gem1000).

**Table 3:** Treatment-related adverse events occurring in ≥10 % of overall patient population

Adverse event (*n* %)	Paz400/Gem1000 (*n* = 6)	Paz800/Gem1000 (*n* = 3)	Paz800/Gem1250 (*n* = 13)	Total (*N* = 22)
Fatigue	4 (67)	3 (100)	8 (62)	15 (68)
Neutropenia	4 (67)	1 (33)	8 (62)	13 (59)
Nausea	4 (67)	2 (67)	6 (46)	12 (55)
Decreased appetite	2 (33)	1 (33)	8 (62)	11 (50)
Leukopenia	2 (33)	1 (33)	7 (54)	10 (45)
Thrombocytopenia	3 (50)	1 (33)	5 (38)	9 (41)
Diarrhea	3 (50)	1 (33)	4 (31)	8 (36)
Vomiting	1 (17)	1 (33)	4 (31)	6 (27)
ALT increased	1 (17)	2 (67)	3 (23)	6 (27)
Dysgeusia	2 (33)	3 (100)	1 (8)	6 (27)
Stomatitis	4 (67)	1 (33)	0	5 (23)
Alopecia	0	0	4 (31)	4 (18)
Hair color changes	1 (17)	0	3 (23)	4 (18)
AST increased	1 (17)	1 (33)	2 (15)	4 (18)
Epistaxis	0	1 (33)	3 (23)	4 (18)
Dry skin	1 (17)	1 (33)	1 (8)	3 (14)

*ALT* alanine aminotransferase, *AST* aspartate aminotransferase, *Gem* gemcitabine, *Paz* pazopanib

### Pharmacokinetics

There was considerable variability in plasma concentrations of pazopanib, gemcitabine, and dFdU during the sparse sampling for patients in the dose-escalation phase (Table 4). Given this variability and the relatively small number of patients enrolled in each cohort, dose proportionality could not be reasonably assessed. In addition, elevations in plasma levels of pazopanib, gemcitabine, or dFdU were not associated with occurrence of either of the 2 DLTs noted above (i.e., Grade 4 thrombocytopenia reported on Cycle 1 Day 8 for one patient in the Paz400/Gem1000 group and Grade 3 fatigue on Cycle 1 Day 1 for one patient in the Paz800/Gem1250 cohort).

**Table 4:** Summary of concentrations of pazopanib, gemcitabine, and dFdU from sparse sampling in the dose-escalation phase

Dose cohort	Cycle day	Nominal time	*N*	Median concentration (range)
Pazopanib concentrations (μg/mL)				
Paz400/Gem1000	C1D1	3.5 h	6	22.5 (6.5–42.8)
	C1D8	Pre-dose	6	22.3 (10.4–37.1)
	C2D1	Pre-dose	6	21.6 (1.0–30.8)
Paz800/Gem1000	C1D1	3.5 h	3	51.0 (36.0–52.1)
	C1D8	Pre-dose	3	24.9 (11.9–52.4)
	C2D1	Pre-dose	3	22.9 (10.9–80.3)
Paz800/Gem1250	C1D1	3.5 h	7	20.7 (7.6–43.3)
	C1D8	Pre-dose	6	23.9 (8.6–37.6)
	C2D1	Pre-dose	4	19.1 (7.4–25.1)
Gemcitabine concentrations (ng/mL)^a^				
Paz400/Gem1000	C1D1	0.5 h	6	10,532 (1,318–11,647)
	C1D8	0.5 h	6	9,691 (994–25,529)
Paz800/Gem1000	C1D1	0.5 h	3	10,820 (9,375–13,881)
	C1D8	0.5 h	3	18,936 (7,913–22,638)
Paz800/Gem1250	C1D1	0.5 h	7	17,854 (8,130–22,541)
	C1D8	0.5 h	6	16,006 (5,274–21,032)
dFdU concentrations (ng/mL)				
Paz400/Gem1000	C1D1	0.5 h	6	29,672 (15,654–54,539)
	C1D8	Pre-dose	6	301 (0–997)
	C1D8	0.5 h	6	31,005 (17,927–38,586)
Paz800/Gem1000	C1D1	0.5 h	3	41,032 (22,844–43,207)
	C1D8	Pre-dose	3	743 (686–1,745)
	C1D8	0.5 h	3	40,789 (24,614–41,044)
Paz800/Gem1250	C1D1	0.5 h	7	34,175 (21,999–45,346)
	C1D8	Pre-dose	6	889 (0–1,678)
	C1D8	0.5 h	6	35,378 (27,767–49,545)

*C* cycle, *D* day, *dFdU* 2′,2′-difluorodeoxyuridine, *Gem* gemcitabine, *h* hour, *Paz* pazopanib

^a^C1D8 pre-dose concentrations for gemcitabine were all less than lower limit of quantitation (50 ng/mL)

**Fig. 1:**
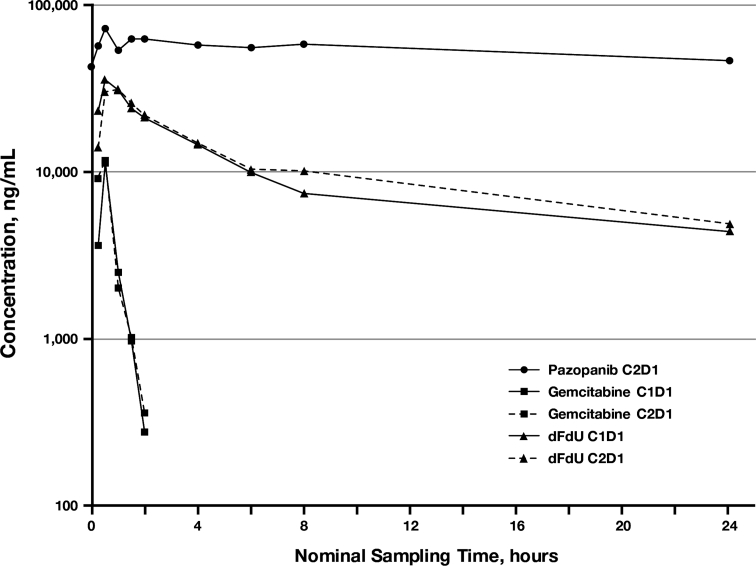
Median pazopanib, gemcitabine, and dFdU concentration–time profiles from patients in the cohort-expansion phase: gemcitabine (1,250 mg/m^2^ by 30-min infusion) was administered alone on Cycle 1 Day 1 (C1D1) and in combination with oral pazopanib (800 mg) on Cycle 2 Day 1 (C2D1); pazopanib was administered once daily beginning on C1D2, and gemcitabine was administered on Days 1 and 8 of the 21-day cycle

Six patients in the cohort-expansion phase had frequent sampling performed for pharmacokinetic analysis. Median concentration–time profiles for pazopanib (Cycle 2 Day 1) and for gemcitabine and dFdU after dosing of gemcitabine alone (Cycle 1 Day 1) and in combination with pazopanib (Cycle 2 Day 1) are presented in Fig. 1. The median gemcitabine and dFdU concentration profiles from gemcitabine alone and from gemcitabine after 21 days of pazopanib administration appear very similar. Gemcitabine is rapidly transformed into dFdU which quickly achieves appreciably greater concentrations with a much longer elimination half-life. Summary pharmacokinetic parameters for pazopanib, gemcitabine, and dFdU are presented in Table 5, along with statistical results from the ANOVA investigating the effect of pazopanib on gemcitabine pharmacokinetics. Because of missed samples, not all pharmacokinetic parameters could be estimated for all patients. Results from the statistical analysis suggest that gemcitabine and dFdU systemic exposures are slightly higher with pazopanib co-administration, with *C*_max_ and AUC geometric least squares mean ratios of 1.06 (90 % CI: 0.62, 1.84) and 1.26 (90 % CI: 0.79, 2.01), respectively, for gemcitabine, and ratios of 0.95 (90 % CI: 0.90, 1.00) and 1.21 (90 % CI: 1.08, 1.36), respectively, for dFdU. This overall assessment is limited by both pharmacokinetic variability and small sample size.

**Table 5:** Summary of pharmacokinetic parameter estimates and statistical results for pazopanib, gemcitabine, and dFdU from the cohort-expansion phase

PK Parameter	Pazopanib	Gemcitabine			dFdU		
	C2D1^a^	C1D1^a^	C2D1^a^	GLM Ratio (90 % CI)^b^	C1D1^a^	C2D1^a^	GLM Ratio (90 % CI)^b^
*C*_max_^c^	73.3 (5) 31.8–98.8	17,380 (4) 10,623–28,664	18,802 (4) 9,175–33,898	1.06 (0.62, 1.84)	36,134 (6) 32,129–44,210	33,911 (5) 28,491–39,942	0.95 (0.90, 1.00)
*T*_max_ (h)	2 (5) 1.58–9	0.50 (4) 0.32–0.53	0.52 (4) 0.25–0.57	–	0.67 (6) 0.50–1.02	1.13 (5) 0.57–1.75	–
AUC_(0–24)_ (h*μg/mL)	1,340 (5) 680–1,777	–	–	–	–	–	–
AUC_(0–∞)_ (h*ng/mL)	–	9,953 (4) 6,522–19,678	11,306 (4) 7,025–21,471	1.26 (0.79, 2.01)	333,145 (6) 244,765–393,908	350,950 (5) 306,839–547,673	1.21 (1.08, 1.36)
*t*½ (h)	47 (4) 32.1–69.5	0.26 (6) 0.26–0.31	0.34 (5) 0.31–0.44	1.28 (1.16, 1.41)	11.9 (6) 10.9–25.7	12.3 (5) 11.4–23.7	1.06 (0.91, 1.24)
CL (L/h/m^2^)	–	135 (4) 64–192	117 (4) 58–178	0.79 (0.50, 1.27)	–	–	–
*C*_min_ (μg/mL)	37.5 (5) 21.5–56.8	–	–	–	–	–	–

*AUC*_*(0-24)*_ area under the concentration–time curve 0–24 h, *AUC*_*(*0–∞*)*_ AUC extrapolated to infinity, *C* cycle, *CI* confidence interval, *CL* clearance, *C*_*max*_ maximum concentration, *C*_*min*_ minimum concentration, *D* day, *dFdU* 2′,2′-difluorodeoxyuridine, *GLM* geometric least squares mean, *h* hour, *PK* pharmacokinetic, *T*_*max*_ time of *C*_max_, *t*_1/2_ elimination half-life

^a^Values denote median (n) and range

^b^Values denote GLM ratio of (C2D1/C1D1) and 90 % CI from analysis of variance

^c^Units for *C*_max_ are μg/mL for pazopanib and ng/mL for gemcitabine and dFdU

### Clinical activity

One partial objective response was initially reported on Day 42 (end of Cycle 2) and sustained through the last assessment on Day 327 in a female patient with melanoma in the Paz800/Gem1250 cohort. Fourteen patients had stable disease at 1 or more disease assessment time points; three of these patients had stable disease for at least 12 cycles (cholangiocarcinoma, 17 cycles; melanoma, 14 cycles; and colorectal cancer, 12 cycles).

## Discussion

This study demonstrated that pazopanib and gemcitabine can be safely administered at doses similar to those given as monotherapy. The most common AEs experienced by patients receiving the combination of pazopanib and gemcitabine were consistent with the known safety profile of each agent individually. The most frequently reported treatment-related AEs were fatigue, neutropenia, nausea, decreased appetite, and thrombocytopenia.

No apparent pharmacokinetic interaction between pazopanib and gemcitabine was observed. However, the assessment was limited by extensive interpatient variability and small sample size. Although this study did not have a period of pazopanib monotherapy without gemcitabine, the pazopanib pharmacokinetic parameters were similar to historical results from pazopanib 800 mg monotherapy [19], suggesting no apparent effect of gemcitabine on pazopanib pharmacokinetics. The pharmacokinetic parameters for gemcitabine and dFdU from both gemcitabine alone (Cycle 1 Day 1) and in combination with pazopanib (Cycle 2 Day 1) were also similar to historical estimates [20, 21].

Preliminary clinical activity was characterized by one patient with a partial objective response and 14 patients with stable disease, including three patients in whom the duration of stable disease ranged from 12 to 17 treatment cycles (21-day cycles).

In summary, therapeutic doses associated with efficacy of both pazopanib and gemcitabine monotherapy were achieved. There was no apparent pharmacokinetic interaction at the highest dose level tested (Paz800/Gem1250), although interindividual variability and small sample size limit the robustness of this inference. The combination of pazopanib and gemcitabine was generally well tolerated, and Phase 2 studies of this combination are warranted.

## References

[1] Sternberg CN, Davis ID, Mardiak J (2010). Pazopanib in locally advanced or metastatic renal cell carcinoma: results of a randomized phase III trial. J Clin Oncol.

[2] van der Graaf WT, Blay JY, Chawla SP (2012). Pazopanib for metastatic soft-tissue sarcoma (PALETTE): a randomised, double-blind, placebo-controlled phase 3 trial. Lancet.

[3] Altorki N, Lane ME, Bauer T (2010). Phase II proof-of-concept study of pazopanib monotherapy in treatment-naive patients with stage I/II resectable non-small-cell lung cancer. J Clin Oncol.

[4] Bible KC, Suman VJ, Molina JR (2010). Efficacy of pazopanib in progressive, radioiodine-refractory, metastatic differentiated thyroid cancers: results of a phase 2 consortium study. Lancet Oncol.

[5] Friedlander M, Hancock KC, Rischin D, Messing MJ, Stringer CA, Matthys GM, Ma B, Hodge JP, Lager JJ (2010). A Phase II, open-label study evaluating pazopanib in patients with recurrent ovarian cancer. Gynecol Oncol.

[6] Sleijfer S, Papai Z, Le Cesne A (2007). Phase II study of pazopanib (GW786034) in patients (pts) with relapsed or refractory soft tissue sarcoma (STS): EORTC 62043 (abstr 10031). J Clin Oncol.

[7] Monk BJ, Mas Lopez L, Zarba JJ (2010). Phase II, open-label study of pazopanib or lapatinib monotherapy compared with pazopanib plus lapatinib combination therapy in patients with advanced and recurrent cervical cancer. J Clin Oncol.

[8] Burris HA, Moore MJ, Andersen J (1997). Improvements in survival and clinical benefit with gemcitabine as first-line therapy for patients with advanced pancreas cancer: a randomized trial. J Clin Oncol.

[9] Carmichael J, Possinger K, Phillip P, Beykirch M, Kerr H, Walling J, Harris AL (1995). Advanced breast cancer: a phase II trial with gemcitabine. J Clin Oncol.

[10] Lund B, Hansen OP, Neijt JP, Theilade K, Hansen M (1995). Phase II study of gemcitabine in previously platinum-treated ovarian cancer patients. Anticancer Drugs.

[11] Sandler AB, Nemunaitis J, Denham C (2000). Phase III trial of gemcitabine plus cisplatin versus cisplatin alone in patients with locally advanced or metastatic non-small-cell lung cancer. J Clin Oncol.

[12] Maki RG, Wathen JK, Patel SR (2007). Randomized phase II study of gemcitabine and docetaxel compared with gemcitabine alone in patients with metastatic soft tissue sarcomas: results of sarcoma alliance for research through collaboration study 002 [corrected]. J Clin Oncol.

[13] Abbruzzese JL, Grunewald R, Weeks EA (1991). A phase I clinical, plasma, and cellular pharmacology study of gemcitabine. J Clin Oncol.

[14] Martin C, Pollera CF (1996). Gemcitabine: safety profile unaffected by starting dose. Int J Clin Pharmacol Res.

[15] Gaitskell K, Martinek I, Bryant A, Kehoe S, Nicum S, Morrison J (2011). Angiogenesis inhibitors for the treatment of ovarian cancer. Cochrane Database Syst Rev.

[16] Ulahannan SV, Brahmer JR (2011). Antiangiogenic agents in combination with chemotherapy in patients with advanced non-small cell lung cancer. Cancer Invest.

[17] Wagner AD, Arnold D, Grothey AA, Haerting J, Unverzagt S (2009) Anti-angiogenic therapies for metastatic colorectal cancer. Cochrane Database Syst Rev 3:CD005392 10.1002/14651858.CD005392.pub3 PMC12227179 19588372

[18] Therasse P, Arbuck SG, Eisenhauer EA (2000). New guidelines to evaluate the response to treatment in solid tumors. European Organization for Research and Treatment of Cancer, National Cancer Institute of the United States, National Cancer Institute of Canada. J Natl Cancer Inst.

[19] Goh BC, Reddy NJ, Dandamudi UB (2010). An evaluation of the drug interaction potential of pazopanib, an oral vascular endothelial growth factor receptor tyrosine kinase inhibitor, using a modified Cooperstown 5 + 1 cocktail in patients with advanced solid tumors. Clin Pharmacol Ther.

[20] Eli Lilly and Company (2012) Gemzar (gemcitabine for injection) powder prescribing information. Revised February 2011 [cited January 5, 2012]. Available from: http://pi.lilly.com/us/gemzar.pdf

[21] Jiang X, Galettis P, Links M, Mitchell PL, McLachlan AJ (2008). Population pharmacokinetics of gemcitabine and its metabolite in patients with cancer: effect of oxaliplatin and infusion rate. Br J Clin Pharmacol.

